# Entrepreneurial nations competing for attention: sport mega-events as strategic assets for soft power

**DOI:** 10.3389/fsoc.2026.1811928

**Published:** 2026-06-25

**Authors:** Faisal AlReshaid, Laila El-Dabt, Shihanah Almutairi, Abrar Al-Enzi, Nour AlBuloushi, Maitham AlSarraf

**Affiliations:** 1 American University of Kuwait, Safat, Kuwait; 2 Australian University, Safat, Kuwait; 3 Abdullah Al Salem University, Khaldiya, Kuwait; 4 Rcyte, Kuwait City, Kuwait

**Keywords:** Abu Dhabi, Dubai, entrepreneurial nations, international relations, soft-power, sports events, strategic management, sustainable nation branding

## Abstract

**Introduction:**

Sport events have become prominent instruments through which states and city-regions attempt to generate attraction, legitimacy, and international influence. Yet, soft power effects are rarely automatic, and they often vary even within the same national setting. This study examines how sport events are strategically mobilised as entrepreneurial instruments of soft power, nation branding, and public diplomacy within the United Arab Emirates by comparing Abu Dhabi and Dubai as two sub-national cases operating under a shared federal structure.

**Methods:**

The research adopts a qualitative comparative case study design and applies data source triangulation by combining systematic documentary analysis with semistructured interviews involving stakeholders in sport governance and event planning. Data was analysed thematically using a combination of deductive coding informed by the Soft Power Hierarchy framework and inductive coding grounded in the empirical material.

**Results:**

Findings show that sport does not function as a uniform pathway to influence across the UAE. Abu Dhabi’s event strategy appears primarily outward-facing, with sport deployed entrepreneurially to build international visibility, strengthen tourism positioning, and compete for attention in a crowded regional environment. Dubai, by contrast, appears to use sport less as a primary external influence tool and more as a mechanism aligned with internal priorities, commercial activity, and the maintenance of an already consolidated global brand.

**Discussion:**

The paper advances soft power theory by reframing influence as a dynamic process and proposing a Soft Power Cycle that foregrounds audiences as active agents who can reinforce or undermine soft power beyond the point of direct policy intervention.

## 1 Introduction

In recent years, the concept of soft power has become increasingly prominent in discussions of international relations, particularly as states seek to pursue influence through attraction and persuasion rather than coercion (Nye, 2004). This shift reflects broader changes in global politics, where cultural visibility, symbolic representation, and communicative practises play an increasingly important role in shaping international perceptions. At the same time, scholars have noted that soft power remains a contested and often misunderstood concept, with ongoing debates surrounding its sources, mechanisms, and actual effectiveness (Mattern, 2005; Fan, 2008).

Within this evolving landscape, sport has emerged as a highly visible platform through which states attempt to project favourable images, foster goodwill, and engage foreign audiences. Sport events in particular have been described as moments of intensified global attention, capable of placing host destinations under prolonged international scrutiny (Cornelissen, 2007). Whilst first-order mega-events such as the Olympic Games and the FIFA World Cup have received the greatest scholarly attention, recent research suggests that sport events more broadly, including second-order and recurring events, can also function as meaningful instruments of diplomacy and nation branding when strategically aligned with broader policy objectives (Donos and Lowes, 2012; Grix and Brannagan, 2016; Al-Khalifa and Al-Khalifa, 2022; Bianco and Sons, 2023; Brannagan and Reiche, 2025).

The United Arab Emirates represents a particularly instructive context for examining these dynamics. Although frequently treated as a unified national actor, the UAE operates as a federal system in which individual emirates retain substantial autonomy over development strategies, governance structures, and branding initiatives. Abu Dhabi and Dubai, in particular, have pursued highly visible yet distinct approaches to sport event hosting, reflecting differences in strategic priorities, institutional arrangements, and perceived positioning within regional and global hierarchies (Grix, 2010; Ulrichsen, 2023; Chadwick and Anagnostopoulos, 2024; Crossley and Woolf, 2024). These variations suggest that soft power strategies may operate differently not only across states, but also within them.

Drawing on Nye’s conceptualization of soft power and Leonard’s framework of behavioural influence, this study examines how sport events are strategically mobilised as instruments of nation branding and public diplomacy within the UAE. Rather than assuming that sport mega-events inherently generate influence, the study adopts a more contextualised perspective that emphasises strategic intent, audience engagement, and reputational maturity. Nation branding and public diplomacy are understood here as mechanisms through which familiarity, appreciation, and engagement are cultivated, potentially creating the conditions under which soft power can emerge (Anholt, 2007; Szondi, 2008; Kaneva, 2011).

Using a comparative qualitative case study of Abu Dhabi and Dubai, informed by documentary analysis and semi-structured interviews with key stakeholders, the paper explores how sport functions differently as a policy tool depending on an actor’s stage of development and international positioning. By focusing on sub-national actors within a single federal state, this research contributes to existing literature by highlighting variation that is often obscured in state-centric analyses of sport diplomacy and soft power. In doing so, it extends current debates beyond first-order mega-events and demonstrates how second-order and recurring sport events may play a sustained role in shaping international perceptions and relationships over time.

## 2 Literature review

### 2.1 Soft power as a contested form of influence

Power has traditionally been treated as the currency of world politics, with states often relying on hard power, including economic and military coercion, to secure desired outcomes (Trunkos, 2013; Pallaver, 2011; Mattern, 2005). Yet global interdependence, growing cross-border visibility, and communication technologies have increasingly challenged the assumption that coercion is the only effective route to influence (Mattern, 2005). In this context, soft power has gained prominence as a strategy built on attraction and persuasion rather than force (Nye, 2004). At the same time, soft power remains debated in the literature, including disagreements over its definition, scope, and how it operates in practise (Fan, 2008; Gallarotti, 2022; Mattern, 2005; Nye, 2021). In contrast to hard power, which is commonly associated with military intervention, coercive diplomacy, and sanctions, soft power is typically grounded in the perceived attractiveness of a state’s culture, its political values, and its foreign policy orientation (Nye, 2004; Gallarotti, 2021).

Nye (2004) positions culture as a central soft power resource, distinguishing popular culture that travels through mass entertainment from “high culture” that transmits ideas and values through education, literature, and the arts. Soft power also draws on political and moral values. Vuving (2009) argues that values can position a country as a symbol of an ideal, which can generate admiration and voluntary alignment. However, value alignment does not automatically translate into power. Instead, values can function as a basis for legitimacy and acceptance, whilst perceived gaps in rights or justice may undermine attractiveness and credibility.

Foreign policy is often treated as the third pillar. It can be understood as a state’s international goals and the strategies used to pursue them (Northedge, 1968). With globalisation increasing the need for cooperation, states have increasingly pursued policies aimed at shaping outcomes through coordination, credibility, and legitimacy rather than coercion (McClory, 2015). These developments help explain why softer mechanisms, including aid or partnerships, may be experienced as more legitimate and less destabilising than overt coercion.

Despite Nye’s wide influence, soft power has been criticised as vague and difficult to operationalise (Ferguson, 2003; Mattern, 2005; Lock, 2010). Critics argue that its underlying “resources” are so expansive that the concept risks meaning everything and therefore nothing (Hoagland, 2004). Yet the breadth of soft power resources can also be seen as a strategic advantage, particularly for small states, because it expands the available repertoire beyond military and financial capacity (Gallarotti, 2023). The practical challenge is not whether resources exist, but whether they can be mobilised effectively through strategy, credibility, and consistent execution (van Ham, 2010).

### 2.2 From resources to outcomes: operationalising influence through the soft power hierarchy

A recurring limitation in soft power debates concerns mechanism. Nye’s framework offers a strong starting point but is less explicit about how soft power is acquired and translated into influence (Grix and Brannagan, 2016). One way to address this gap is to treat soft power not as an inherent quality of resources, but as a strategic process shaped by objectives, audiences, and sequencing. Goal-setting has long been treated as foundational for strategy development (Mitchell, 2004). In soft power terms, McPherson et al. (2017) similarly begin with defining desired outcomes and identifying the audiences whose behaviours matter. Leonard (2002) clarifies this behavioural dimension by showing how objectives such as attracting tourism, investment, or support for national positions rely on voluntary action, not coercion.

Leonard (2002) proposes three building blocks of behavioural influence. First, states must build familiarity, because audiences encounter vast volumes of information and retain only a small fraction. This encourages the use of simplified, repeated messages that generate recognition and recall. Second, states must cultivate appreciation by shaping positive perceptions and explaining context so that actions are interpreted in ways that reduce backlash or misunderstanding. Third, influence is strengthened through engagement, particularly via two-way exchanges that build trust and relationships. These mechanisms provide a practical bridge between abstract resources and observable outcomes.

**Figure 1:**
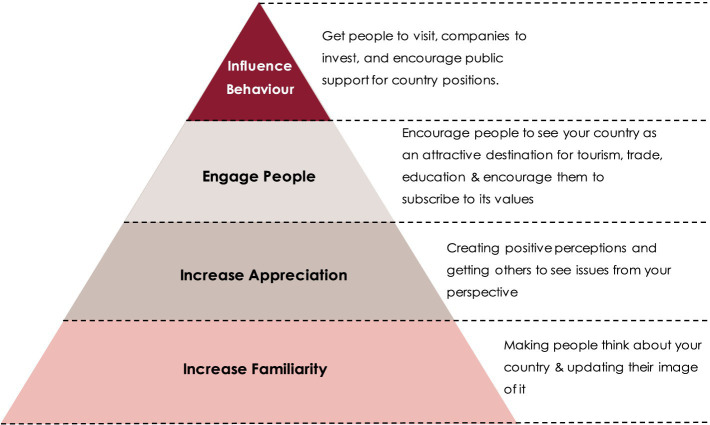
Behavioural influence pyramid. Source: adapted from Leonard (2002).

The pyramid below (Figure 1) has been constructed to visually summarise the hierarchal structure of behavioural influence described by Leonard (2002). Behavioural Influence Pyramid will be adapted and re-coined as the Soft Power Hierarchy throughout the remainder of this study.

### 2.3 Nation branding as structured familiarity and early-stage appreciation

Nation branding emerges from the premise that countries, like corporations, operate in reputational markets where perception shapes opportunity (Anholt, 1998). It is often defined as the application of marketing concepts to countries to enhance reputation in international relations (Kerr and Wiseman, 2013). Whilst some critics treat nation branding as little more than slogan-based persuasion, others argue that it can operate as a broader strategic process rather than propaganda (Kaneva, 2011). Across the literature, a recurring thread is that nation branding seeks to increase visibility, shape image and identity, and establish a competitive identity in ways that support political and economic interests (AlSarraf and Almutairi, 2025; Fan, 2006; Gudjonsson, 2005; Anholt, 2007; Szondi, 2008; Kaneva, 2011).

A key practical contribution of nation branding is increasing awareness, which is particularly relevant for small and mid-sized states seeking recognition and legitimacy in crowded global environments (Keller, 1993; Stokes, 1985). Branding strategies also often aim to reduce the gap between brand identity (intended meaning) and brand image (external perception). Beyond general visibility, competitive identity frameworks propose that states may differentiate across domains such as tourism, exports, governance, investment, culture, and people (Anholt, 2005). Because resources are finite, some states prioritise niche segments and develop sub-brands aligned to functional contexts such as tourism, business, or sport (Murtha and Lenway, 1994; Knott et al., 2015).

In terms of communication form, nation branding is typically anchored in one-way messaging that targets mass audiences through slogans, visuals, and promotional campaigns (Szondi, 2009; Fan, 2010). Messages are often repeated to build recall and top-of-mind associations, and they tend to foreground positive and marketable aspects of national identity (Szondi, 2008; Leonard, 2002). Within the Soft Power Hierarchy, this positions nation branding primarily within familiarity and, to some extent, early appreciation. It rarely reaches the engagement layer because its communication model leaves limited room for dialogue.

### 2.4 Public diplomacy as strategic understanding and engagement

Public diplomacy is typically distinguished from traditional diplomacy by its audience and channels. Traditional diplomacy focuses on state-to-state negotiations, whereas public diplomacy targets publics, groups, and non-state actors in foreign societies (Melissen, 2005; Lee and Hocking, 2011; Keukeleire, 2003). Early definitions emphasised influencing foreign governments indirectly through public opinion (Delaney, 1968; Malone, 1988). More recent interpretations emphasise strategic communication aimed at shaping a receptive environment for national interests by improving understanding, reducing resistance, and supporting long-term legitimacy (Szondi, 2008; Carter Review, 2005).

Public diplomacy often operates through two complementary modes. One mode relies on one-way communication to explain policy decisions, reduce misunderstanding, and provide context that helps publics interpret state actions more favourably. A second mode relies on two-way engagement, including exchanges and dialogue, to build trust and durable relationships. Compared with nation branding, public diplomacy is typically more targeted, more sensitive to audience segmentation, and more attentive to timing and context. As a result, it can reach deeper layers of the Soft Power Hierarchy, extending from appreciation into engagement.

### 2.5 Linking the mechanisms to soft power: towards a dynamic model

Taken together, the literature suggests that soft power is better understood as a process rather than a static attribute. The existence of cultural resources, political values, or foreign policy narratives does not automatically translate into influence. Instead, influence depends on whether these resources are mobilised strategically and communicated in ways that shape how external audiences interpret a nation, how they feel towards it, and whether they are willing to engage with it over time (Nye, 2004; Leonard, 2002). In this sense, nation branding and public diplomacy function as operational pathways through which soft power may emerge, rather than as soft power in themselves.

Within this process, nation branding is most closely associated with building familiarity and early-stage appreciation through one-way communication that increases recognition and establishes competitive identity claims (Anholt, 2007; Fan, 2010). Public diplomacy, by contrast, extends the pathway into deeper appreciation and engagement through explanatory communication and, more importantly, two-way exchanges that facilitate trust-building and long-term relationship formation (Melissen, 2005; Szondi, 2008). This distinction matters because soft power outcomes depend not only on whether audiences notice a nation, but also on whether they come to view it as credible, legitimate, and worth engaging with.

**Figure 2:**
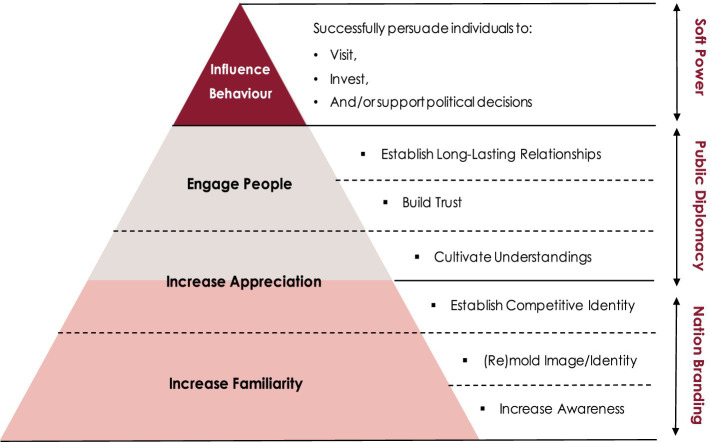
Soft power hierarchy. Source: El-Dabt et al. (2025).

To integrate these mechanisms more clearly, the present framework positions nation branding and public diplomacy within the familiarity, appreciation, and engagement stages of the Soft Power Hierarchy (El-Dabt et al., 2025). Figure 2 summarises this relationship by locating nation branding primarily within familiarity and appreciation, whilst locating public diplomacy across appreciation and engagement. This framing provides conceptual clarity and helps avoid a common limitation in the literature, where nation branding, public diplomacy, and soft power are used interchangeably despite reflecting different levels of influence.

Table 1 clarifies the conceptual boundaries between nation branding and public diplomacy by summarising their communication logics, interaction depth, and intended outcomes. This distinction is necessary because both rely on image and communication practises, yet they differ in temporal orientation and capacity to generate sustained relational effects.

**Table 1:** Summary of conceptual characteristics.

Conceptual characteristic	Nation branding	Public diplomacy	Soft power
Intended outcomes	Enhance awareness, (re)mold image/identity, promote competitive advantage	Cultivate understandings, foster trust, build long-lasting relationships	Persuade and influence behaviours
Intended perception	Be seen as relevant, unique, attractive, exciting	Be seen as similar, respectful, admirable, and trustworthy	Be seen as all aforementioned characteristics
Link to soft power resources/Pillars	Promotes positive marketable “cultural” elements	Addresses positive and negative foreign policies and political morals/values	Culture, foreign policy, political morals and values
Operates at	Level of familiarity and appreciation through awareness/top-of-mind associations	Level of appreciation and engagement through fostering understandings and relationships	Level of attitudinal or behavioural influence
Audience	Mass, homogenous audience	Targeted, segmented geopolitical groups, countries, etc.	Typically segmented according to objective but can be mass
Channels	One-way	One-way when informing, two-way when engaging	One-way and Two-way
Content	Simplistic messages	Complex messages	Simple and complex behaviours
Context	Times of peace	Times of Conflict and Peace	Times of conflict and peace
Examples of initiatives	Plastering of country logos, slogans, and advertisements	Cultural exhibitions, exchange programmes, conferences, politicised media channels	Combination of NB and PD initiatives that led to tourism/investments/political support

Nation Branding + Public Diplomacy leads to Soft Power.

Source: author’s own work.

Nation branding is the repetitive promotion of simplistic messages, images and information directed at mass audiences to increase familiarity and generate top-of-mind associations regarding a country’s existence, image, identity, and/or competitive advantage (El-Dabt et al., 2025).

Public diplomacy is the strategic communication and engagement process which conveys complex messages tailored towards target audiences to cultivate understandings, build trust and credibility and foster relationships between governments and foreign publics (El-Dabt et al., 2025).

### 2.6 Sport mega events (SME) and the soft power pathway

Sport mega-events have increasingly been linked to soft power because they generate concentrated international visibility and symbolic representation, often over extended periods (Cornelissen, 2007; Dubinsky, 2019; Santos, 2021). At the familiarity level, events are frequently described as “coming-out parties” that place hosts on the map, increase visibility, and shape baseline recognition (Cornelissen, 2007; Rein and Shields, 2007; Grix et al., 2024). Events can also provide opportunities to reframe negative images or contest stereotypes, particularly when hosts manage symbolic and operational aspects carefully (Grix and Lee, 2013; Horne and Manzenreiter, 2006; El-Nawawy and Elmasry, 2024; Karlsson and Wenell, 2026).

Beyond familiarity, events can support appreciation by projecting competitive identity and offering richer narratives than slogans alone. They may also function as public diplomacy platforms through speeches, media coverage, conferences, and curated messaging that provide more contextual understandings of a host’s social, political, or cultural positioning (Olins, 2002). At the engagement level, events bring stakeholders into contact across multiple layers, including officials, sponsors, organisers, media, and visiting publics. These interactions can generate trust and credibility when experience aligns with messaging, and they can also produce reputational damage when misalignment generates perceptions of manipulation or ‘soft disempowerment’ (Brannagan and Giulianotti, 2015; Horne and Manzenreiter, 2006; Grix and Brannagan, 2016; Chadwick and Anagnostopoulos, 2024; Crossley and Woolf, 2024; Grix et al., 2025).

Whilst hosting an event does not automatically change a state’s culture, values, or foreign policy, it can create visible demonstrations of competence, goodwill, and reliability that support credibility and trust (Donos and Lowes, 2012). When familiarity, appreciation, and engagement are successfully built and sustained beyond the event itself, sport events can contribute to outcomes that rely on voluntary behaviour, including tourism, investment, and political support. In this sense, sport events are best understood as strategic instruments that can activate the soft power pathway, rather than as inherently soft-power-generating phenomena.

Recent scholarship has further complicated the relationship between sport, soft power, and diplomacy by showing that sport-event strategies do not simply project attraction, but are also interpreted, contested, and sometimes resisted by external audiences. Contemporary soft power research has therefore moved beyond treating attraction as a resource and has placed greater emphasis on reception, credibility, and the distinction between resources, instruments, and outcomes (Henne, 2022; Ohnesorge, 2020). In sport-focused research, this has been especially evident in debates on sport diplomacy, sportswashing, soft disempowerment, and the use of mega-events by Gulf states to pursue visibility, legitimacy, and geopolitical positioning (Brannagan and Giulianotti, 2018; Dubinsky, 2019; Al-Khalifa and Al-Khalifa, 2022; Bianco and Sons, 2023; Ulrichsen, 2023; Grix and Brannagan, 2024; Grix et al., 2025). This literature reinforces the need to treat soft power not as an automatic outcome of hosting, but as a contingent process shaped by audience interpretation, credibility, message-experience alignment, and regional competition.

## 3 Methodology

This study adopts a qualitative research design grounded in a comparative case study approach. The purpose of this design is to develop a contextualised understanding of how sports events are strategically mobilised as instruments of soft power and nation branding within the United Arab Emirates. Qualitative case study research is particularly appropriate for examining complex, context-dependent phenomena, as it allows for an in-depth exploration of strategic intent, governance rationales, and implementation processes that cannot be adequately captured through quantitative methods (Yin, 2014; Edwards and Holland, 2013).

Unlike methodological triangulation, which involves using different research methods such as survey, observations, and interviews (e.g., AlReshaid et al., 2025, 2026), this study employs data source triangulation by combining documentary analysis with semi-structured interviews. Triangulation enhances credibility by examining the same phenomenon through multiple sources, thereby reducing reliance on any single method or perspective (Patton, 2002; Crowe and Sheppard, 2011).

### 3.1 Case selection

Abu Dhabi and Dubai were purposively selected as the focal cases for this study due to their central roles in shaping the UAE’s sport event landscape. Whilst both emirates operate within the same federal governance structure, they differ substantially in terms of development trajectories, institutional arrangements, and approaches to sport event hosting. These differences provide a meaningful basis for comparative analysis. Examining these two emirates enables the study to investigate how sub-national actors within a single state pursue differentiated soft power and nation branding strategies through sport, whilst remaining embedded within a shared national and cultural context (Grix, 2010; Yin, 2014). The study is also deliberately framed as a historical analysis of the 2017–2018 period, when the interviews were conducted and when Abu Dhabi and Dubai were consolidating distinct sport-event strategies. The findings should therefore be read as capturing stakeholder interpretations during this specific phase of UAE sport-event development, rather than as a current inventory of the sport-event landscape in 2026.

### 3.2 Document analysis

The first phase of data collection involved a systematic documentary analysis aimed at identifying strategic priorities, policy narratives, and branding objectives associated with sport event hosting in each emirate. Documentary analysis is a well-established qualitative method that supports the interpretation of meaning, the development of contextual understanding, and the identification of institutional intent (Bowen, 2009).

The document corpus included national and emirate-level vision statements, economic and tourism strategies, sport sector policies, and official government publications. In cases where sport-specific strategies were not publicly available, particularly in Abu Dhabi, supplementary materials such as authoritative media reports and published interviews with senior officials were incorporated. Document selection prioritised relevance, institutional credibility, and temporal alignment with major sport event hosting phases (Bowen, 2009).

Although policy documents provide essential strategic context, they are inherently forward-looking and static. To address this limitation and capture evolving interpretations and practises, the documentary analysis was complemented by primary interview data, as recommended in qualitative policy research (Patton, 2002).

### 3.3 Semi-structured interviews

The second phase of data collection consisted of semi-structured interviews with stakeholders directly involved in sport governance, event organisation, and strategic planning. Semi-structured interviews are particularly effective for eliciting detailed insights whilst maintaining flexibility to explore issues raised by participants (Bryman, 2008; Edwards and Holland, 2013).

Interview participants included representatives from emirate-level sports councils, national sport governing bodies, and organising committees responsible for major sport events hosted in Abu Dhabi and Dubai, as summarised in Table 2. These interviews provided valuable insight into event selection criteria, strategic priorities, and perceptions regarding the role of sport events in advancing soft power and nation branding objectives.

**Table 2:** Summary of interview participants and anonymised quotation codes.

Code	Emirate	Institutional affiliation	Role category/relevance to study	Interview date
D1DSC	Dubai	Dubai Sports Council	Senior sport-governance stakeholder involved in Dubai sport strategy development	April 2, 2018
D2DSC	Dubai	Dubai Sports Council	Sport-governance stakeholder involved in event coordination and institutional support	April 2, 2018
D3DSC	Dubai	Dubai Sports Council	Sport-governance stakeholder involved in event selection, tourism positioning, and local sport activity	April 2, 2018
D4MH	Dubai	Meydan Holding/Dubai World Cup	Event-management stakeholder involved in Dubai World Cup strategy and brand positioning	April 3, 2018
D5MH	Dubai	Meydan Holding/Dubai World Cup	Event-management stakeholder involved in Dubai World Cup organisation and commercial objectives	April 3, 2018
D6MH	Dubai	Meydan Holding/Dubai World Cup	Event-management stakeholder involved in Dubai World Cup operations and audience experience	April 3, 2018
D7EC	Dubai	Dubai Executive Council	Strategic planning stakeholder involved in Dubai wider development strategy	April 3, 2018
AD1ADSC	Abu Dhabi	Abu Dhabi Sports Council	Sport-governance stakeholder involved in events, marketing, communications, and statistics	July 11, 2017
AD2ADSC	Abu Dhabi	Abu Dhabi Sports Council	Sport-governance stakeholder involved in branding, tourism positioning, and event strategy	July 11, 2017
AD3ADSC	Abu Dhabi	Abu Dhabi Sports Council	Sport-governance stakeholder involved in government coordination and tourism-related event strategy	July 11, 2017
AD4F1	Abu Dhabi	Abu Dhabi Formula One	Event-management stakeholder involved in Formula One event strategy and visitor experience	July 12, 2017

Source: Author’s own work.

### 3.4 Data collection and analysis procedure

The documentary corpus consisted of 13 core documents and supplementary sources: seven related primarily to Dubai and six related primarily to Abu Dhabi. For Dubai, the main documents reviewed were the Dubai Strategic Plan 2015, Dubai Plan 2021, Dubai Plan 2021 Strategic Report, Dubai Sports Council strategy materials for 2007–2010 and 2011–2015, publicly available reporting on the 2016–2020 Dubai Sports Council strategy, and tourism and industry reports addressing Dubai’s sport and visitor economy as shown in Appendix A. For Abu Dhabi, the main documents reviewed were the Abu Dhabi Plan/2030 vision materials, Abu Dhabi Economic Vision 2030, Abu Dhabi tourism and visitor reports, Abu Dhabi Council for Economic Development materials, Abu Dhabi Sports Council-related press coverage, and industry reports discussing major events such as Formula One, golf, cycling, sailing, and air racing. Because Abu Dhabi’s sport-specific strategies were not publicly available, authoritative media and industry sources reporting interviews with senior officials were used to supplement official documents.

Documents were selected according to three criteria: relevance to sport events, tourism, economic development, nation branding, public diplomacy; institutional credibility; and temporal alignment with the sport-event strategies examined in the study. In assessing each document, attention was paid to the authoring institution, the purpose of the document, the intended audience, and the publication date.

Data analysis followed an iterative thematic procedure across documentary and interview materials (Spencer et al., 2003; Braun and Clarke, 2006). Coding was conducted through three rounds. The first round involved familiarisation, during which documents and interview transcripts were read repeatedly and initial notes were made on recurring strategic priorities, event rationales, and policy narratives. The second round used inductive coding to identify themes emerging from the data, such as commercial logic, local entertainment, landscape showcasing, regional competition, institutional fragmentation, tourism positioning, and reputational maturity (Crabtree and Miller, 1999). The third round reconciled these inductive codes with deductive categories derived from the Soft Power Hierarchy and related nation branding and public diplomacy scholarship, including familiarity, appreciation, engagement, nation branding, public diplomacy, strategic visibility, and behavioural influence (Leonard, 2002; Grix and Brannagan, 2016). Themes were then refined through constant comparison across documents and interviews to identify patterns that were shared across sources and those that were specific to Abu Dhabi or Dubai, thereby strengthening coherence and triangulated validity (Gale et al., 2013).

### 3.5 Ethical considerations

Ethical approval was obtained prior to data collection. All interview participants were informed of the purpose of the study and assured of confidentiality and anonymity. Informed consent was obtained before each interview. To protect participant identities, a coding system was used in reporting findings, and no personally identifiable information was disclosed. All data were securely stored and used exclusively for academic research purposes, in line with established qualitative research ethics (Bryman, 2008).

## 4 Results

### 4.1 Document analysis

The UAE’s federal structure grants emirates substantial autonomy over governance and development strategy. Because Dubai and Abu Dhabi have been the primary drivers of sport-event policy and international positioning within the federation, this section compares their long-term visions and sport strategies, rather than treating the UAE as a single undifferentiated actor.

#### 4.1.1 Overview of Dubai’s strategies

In February of 2007, Dubai launched its Strategic Plan which outlined its growth strategy till 2015, after which another plan paving the way to 2021 was launched and one update report was released in early 2017. In contrast to all other long-term strategies within the Gulf which were launched at the end of 2008 and were meant to guide the countries till 2030, Dubai’s strategies covered a much shorter time-span. This could be attributed to the rate of development and progressive state of the emirate in contrast to other places in the Gulf, including their local neighbours Abu Dhabi.

With respect to sports strategies, these documents were also constructed to cover shorter timespans. The first sport strategy covered the years 2007–2010, whilst the second one covered the years 2011–2015. Although Dubai has also launched a 2016–2020 strategy, this document was not made publicly available as were the first two. However, brief details regarding this strategy were unveiled in an interview with the Secretary General of the Dubai Sports Council covered by Gulf News.

The introductory remarks of the 2021 plan referenced the success of the previous strategy for driving Dubai forward in various sectors including trade, financial services, logistics and tourism, and highlighted that the newly found strategy will build upon this success to continue to:

‘Propel Dubai to be amongst the world’s greatest cities, and to reinforce its position as a pivotal hub in the global economy as well as the preferred place to live and work for people and visitors alike’ (Executive Council of Dubai, 2015; p. 2).

Drawing on the success of its previous 2007 strategy, the 2021 plan is focused on reaffirming a more definitive positioning of the emirate as a global economic hub and enhancing its internal environment to ensure that it is the world’s preferred destination to live, work and visit.

Although “preference” is inherently subjective, Dubai Plan 2021 operationalises liveability and global competitiveness through six governance themes: citizen wellbeing and empowerment; social cohesion; destination attractiveness for living, working, and visiting; smart sustainability; global economic hub positioning; and government excellence. Within the scope of this study, the plan’s relevance lies less in the full thematic architecture and more in three overlapping emphases that intersect with the Soft Power Hierarchy: (1) consolidating Dubai’s identity as a global economic hub, (2) sustaining its attractiveness as a place to live, work, and visit, and (3) projecting values of tolerance and inclusion as part of a stable internal environment.

Prior to delving into these themes, it is important to note Dubai makes no mention of establishing a top sporting industry nor using sport as a primary tool for development. Instead, sport plays a complementary role in the emirate’s strategy and certain initiatives cross-relate with sport or have “spill-over” effects to benefit some of the predetermined objectives as will now be discussed.

Dubai’s strategic trajectory has been closely tied to tourism-led diversification and the deliberate cultivation of an internationally legible destination brand. Early institutional efforts, such as the establishment of the Dubai Commerce and Tourism Promotion Board, explicitly targeted affluent leisure and business segments (Stephenson and Ali-Knight, 2010; p. 279). By 2018, Dubai’s scale and commercial maturity were reflected in high tourist volumes and strong visitor expenditure performance (Euromonitor International, 2018; Mastercard Report, 2018). Within Dubai Plan 2021, this positioning is further consolidated through an explicit objective to reinforce Dubai’s standing as a global centre for trade, logistics, finance, and tourism (Executive Council of Dubai, 2015; p. 17).

Dubai’s tourism identity functions both as a component of, and a reinforcement mechanism for, its broader “global hub” brand: sustained visibility campaigns and signature urban developments have helped translate recognition into routinised visitation behaviours. In Dubai’s own strategic reporting, international conferences, exhibitions, and landmark projects are presented as key contributors to this reputational consolidation (Executive Council of Dubai, 2017).

Dubai Plan 2021 also extends the tourism narrative into a broader competitiveness frame by foregrounding a business-friendly environment and initiatives such as Dubai’s positioning as the “Capital of Islamic Economy” (Executive Council of Dubai, 2015; p. 17). In this context, sport is framed less as a standalone development pillar and more as an enabling sector that can support tourism flows and commercial diversification rather than define the emirate’s core strategic identity (ibid).

As highlighted in the overarching mission, the 2021 strategy aims to make Dubai the preferred place to live, work and visit. Whilst this point can relate to the image/identity positioning discussed in the previous section, it differs as it extends beyond the promotion of simplistic messages on the international front to attract those from abroad and instead focuses on the establishment of an intrinsic environment that serves those within the nation. Primary initiatives consisted of ensuring a safe and secure environment, the provision of adequate education, healthcare and housing services to foster a ‘happy, creative and empowered’ population. Building on these fundamentals, vibrant cultural experiences that are distinctive to Dubai are meant to ‘improve Dubai’s livability by building on and improving the experiences of locals’ (Executive Council of Dubai, 2015; p. 13).

Once again targeting Dubai’s local population which consists of a highly diverse demographic, the 2021 Plan highlights the importance of establishing a tolerant and inclusive society that celebrates cultural diversity and embraces civic values. By catering to all races, cultures, and physical capabilities of individuals within the nation, it is more likely that the city will achieve its goal of being viewed as a preferred place for a wider range of individuals. Drawing back to the concept of soft power once again, the embodiment of commonly accepted values such as tolerance, justice, and inclusion, can minimise the amount of criticism and resistance faced by a nation, and in turn, lead to favourable views. Dubai does not intend to instil these values to “bridge gaps” between foreign audiences. Instead, this initiative also serves to foster a city of happy, creative and empowered people. Thus, Dubai’s efforts are meant to ensure positive livable environments, rather than establish an understanding of its society.

#### 4.1.2 Overview of Abu Dhabi strategies

In 2008, Abu Dhabi launched its 2030 National Vision. Unlike the pre-discussed strategies, however, it has not launched a comprehensive document to cover its plans. Instead, the overall 2030 vision, and its associated pillars of social development; security, justice and safety; infrastructure and environment; economic development; and government affairs were merely listed on a government-affiliated website. However, a detailed Economic Development strategy was released in 2008 and an official tourism review report was published in 2014 and will therefore be used to provide this section with additional details (see Abu Dhabi Tourism and Culture Authority, 2014; Department of Economic Development, 2008).

Furthermore, due to a lack of publicly available sports strategy documents, this section can only provide insights based on published news articles that discuss the emirate’s sport strategies. In regards to the 2008–2012 plan, an article entitled *Sport in Abu Dhabi to Receive a Boost*, discloses information provided by the Secretary General of Abu Dhabi Sports Council in a press conference. Whereas the 2012–2016 Sports Strategy was briefly covered in a similar manner in the Oxford Business Review report published in 2016 which unveiled findings from an interview with the Secretary General at the time.

Abu Dhabi’s national website outlined the following aim for its 2030 vision:

‘The Abu Dhabi Plan is a reflection of the late Sheikh Zayed bin Sultan Al Nahyan’s legacy; a strategic blueprint that has been designed to guide the Emirate of Abu Dhabi’s growth and development enabling Abu Dhabi to achieve its vision of a secure and confident society that aims to develop a competitive, sustainable and globally open economy’ (Department of Economic Development, 2008).

Based on the review of the official and unofficial documents discussed above, only two primary themes emerged as relevant to this research. These themes were to: (a) increase awareness of Abu Dhabi, and (b) manage the emirate’s reputation as a unique tourism destination. Abu Dhabi has struggled from a lack of awareness particularly since they have been cast in the shadows of their local neighbours, Dubai. The 2030 Vision website does not highlight this particular point of “increasing awareness” but it has explicitly been highlighted in the official Economic Review and by various sporting officials interviewed throughout press releases.

Throughout these reports, it has become clear that the emirate does not shy away from using international events as a way of promoting its name on the global front. Within the Economic Review, The Acting Executive Director of Abu Dhabi Tourism and Culture Authority was cited stating: ‘We want the maximum positive worldwide publicity that we can gain out of [events]… we look for brand association with a global federation or rights-holder, global media coverage and visibility in key and emerging source markets’ (Statistics Centre Abu Dhabi, 2014; p. 16). In addition to evaluating an event’s visibility in target markets, the emirate also strategically assesses whether an event is aligned with showcasing the emirate’s cultural and heritage assets (ibid).

Furthermore, the intention to brand Abu Dhabi through sports events has played a clear role in the Emirate’s strategy from the start. In the article covering a press conference with the Secretary General of the Abu Dhabi Sports Council in 2008, it stated that:

The Abu Dhabi Golf Championship and the Red Bull Air Race have already taken Abu Dhabi to the world, [and] the Abu Dhabi Tourism Authority have also carried ‘Brand Abu Dhabi’ through the World Rally Championship and UIM Formula One powerboat races. With this latest initiative through ADSC, the government is all set to create a few more vehicles to promote both the emirate and the UAE as a major sporting destination.

When scrolling through the infographics and high-level information regarding overarching goals and programmes on the government website, “managing Abu Dhabi’s media reputation and position” and “sports mega-events” emerge under the objective of attracting tourism, which allows one to conclude that these initiatives are viewed as synergistic. The economic review further highlights that Abu Dhabi’s scenic landscape and cultural heritage are misrepresented on the international front and need to be promoted on a broader scale (Abu Dhabi Council for Economic Development, 2014).

Reaffirming these objectives, the tourism section of the 2030 Economic Strategy document begins with the statement that ‘a vibrant business, culture, leisure, and sports segment is being developed… to cater to the growing number of high-end tourists and visitors, as well as the National and resident population’ (Government of Abu Dhabi, 2007; p. 117). The emirate is most definitely known for its abundant wealth, however, it is trying to focus on niche segments that Dubai has not invested in to distinguish itself from its neighbours. Therefore, Abu Dhabi has tried to capitalise on its land space by developing cultural attractions on its islands which consist of clusters of museums, cultural and educational institutions rather than tall lavish skyscrapers like Dubai (ibid). The Tourism Authority has also opened international offices in mature European markets such as the UK, Germany and France to ‘enhance the promotion and marketing of the destination and its brand’ (Government of Abu Dhabi, 2007; p. 117).

These objectives have also driven the mega-event hosting rationale adopted by governing officials as they try to use sport as a niche segment to further promote the emirate’s scenic landscape and cultural heritage. For example, within the press release, the Abu Dhabi Sports Council Secretary General references the Volvo Ocean Race as one that allows the Emirate to demonstrate its local capabilities whilst leveraging its ‘rich maritime heritage’, and the HSBC Golf Championship as one that ‘put Abu Dhabi on the international golf tourism map’. Building on this notion, it was explicitly noted that in an up-and-coming tourist destination like Abu Dhabi, sporting events serve as advertising tools in which the emirate can be “marketed” in a positive manner. The Abu Dhabi Grand Prix has also been identified as a prime example of this motive as its timing can help show those in other countries how Abu Dhabi’s weather is still warm in November, and perhaps entice those living in colder climates to come visit (ibid).

Further building on these objectives, the second Secretary General who was in charge of developing the 2011 sports strategy, stressed the importance of hosting high-profile events. Al-Awani was quoted saying: ‘our aim is to attract a greater volume of sporting events that have international appeal whilst striking a balance between professional and amateur tournaments’ (Oxford Business Group, 2016, p. 272). The emirate is a large advocate of using independent research companies to provide brand measurement and media tracking services to confirm these impacts. The document explicitly credits the Red Bull Air Race, Volvo Ocean Race, the Abu Dhabi Tour, and Formula One as primary platforms.

Abu Dhabi’s event portfolio is repeatedly justified through its capacity to visually narrate place: unlike stadium-bounded events, formats such as air racing, offshore sailing, and multi-stage cycling are treated as moving broadcasts of landscape, infrastructure, and symbolic modernity. Even where competition is spatially fixed (e.g., Formula One), the surrounding entertainment ecosystem is presented as integral to the destination proposition and thus to reputational positioning (Abu Dhabi Council for Economic Development, 2014).

### 4.2 Interviews

A total of 11 interviews were conducted within the UAE; seven of which were from candidates in Dubai and four of which were from candidates in Abu Dhabi. Despite the significantly lower number of interviewees in each destination, their combined expertise allowed them to provide informative answers that were of relevance to this research.

Based on the interviews conducted with stakeholders in different sectors, it was found that Dubai’s sports strategy was initially geared to (a) help promote Dubai as a tourism destination, (b) support commercial interests and facilitate business networks using sports events and (c) provide an entertaining environment for locals.

#### 4.2.1 Promoting Dubai as tourism destination

Having been involved in the construction of each strategy, the Ex-Secretary General of Dubai Sports Council explained that Dubai’s initial strategy was geared towards improving the performance of Dubai’s local sports clubs. He noted that:

Our main goal was to improve the results of our clubs since they were bad at the time. The critical decision of the first report was to start professionalism in Dubai and focus on it. We started with that and created the rules and regulations.

Once these foundations were established and club performances improved, the DSC ensured that its clubs signed with international federations such as FIFA. This allowed them to later capitalise on sports tourism opportunities through hosting affiliated events. When selecting their tournaments however, the Sports Council chose to host events that could showcase Dubai’s “transforming” landscape and improved club performance at the time. As noted by one interviewee, prior to the Palm development the emirate was not very well-known for its beaches (D3DSC, March 26, 2018). Thus, the DSC selected a variety of beach-related tournaments such as beach volleyball and beach football to showcase their landscape.

We tried to link the event itself with Dubai’s goals. At that time, everybody said Dubai did not have many beaches. We already had 70 km of beach but not enough to attract more tourism. A whilst later, the palm was created and there was more than 300 km of beach, so we saw what sports showcase our beaches. We signed with FIFA then and the first tournament we had was beach football (D1DSC, April 2, 2018).

In addition to showcasing Dubai’s landscape, a second criterion in the DSC’s event selection strategy was to target culturally relevant events and use them as a way to attract tourists. Swimming, horseracing and camel racing have been identified as culturally relevant events. Camel racing in particular is considered a unique sport to Arab cultures and one that may intrigue those from Western countries. Thus, it is promoted by the tourism authority and showcased in exhibitions and on websites alongside Dubai’s horseraces which are also linked to Emirati heritage as explained in the following comment:

Later, we started to focus on things close to our culture like swimming so we signed with FINA… It wasn't just for the sake of bringing an event. Even in beach football we opened the stadium, a temporary stadium close to the beach and in the beginning of the tournament it was open for the public. Also, when we participate in exhibitions or conferences we showcase the camel and horseraces because this is also part of our culture. We also promote them on websites (D1DSC, April 2, 2018).

It is important to note that the cultural identity associated with horseracing is not as unique to Gulf culture as camel racing is, but it does have a special significance to the emirate of Dubai as the Sheikh himself has a strong interest in the sport. Thus, Dubai’s hosting of what was then the world’s richest horse race, with prize money of US$12 million before being surpassed by the US$20 million Saudi Cup in 2020, appears to have been driven primarily by Sheikh Mohammed’s personal interest in horseracing rather than by explicit national branding or soft power objectives.

#### 4.2.2 Support commercial interests and facilitate business networks

Throughout the interview process, it was also highlighted that other sport events such as the Rugby Sevens and the Desert Classic golf tournament, are also held by private entities rather than the Dubai Sports Council. Barring interest from the Sheikh, these tournaments are mainly driven by the commercial interests of certain businesses within Dubai and may obtain financial support from the governments or operational support from the DSC when needed. Reaffirming this notion, one candidate expressed:

In relation to cultural sports like horse riding, you can actually consider it a special case. Sheikh Mohammad loves horses and it’s a big investment that he concentrates on himself. So, the Meydan Dubai World Cup can be considered a more private event and there are special offices and parties involved but we [the DSC] help out if they need it (D2DSC, April 2, 2018).

Even with golf for example, we may help arrange with the organisers who have huge investments directly from Dubai governments but these tournaments don’t fall directly under our umbrella; it’s a private entity… but they are considered private tournaments if it’s from his highness or from the private sector (D2DSC, April 2, 2018).

It was surprising to find that the events that Dubai is most famously renowned for (the Emirate’s Rugby Sevens, Meydan Horse World Cup, and the Omega Golf Classic), do not fall under DSC’s strategic plan. Instead, these events are held to generate revenues and promote specific company brands rather than the nation’s brand. Upon gaining this clarification, we delved deeper about any additional criteria behind the DSC’s event selection strategy. It was explained that in addition to showcasing Dubai as a tourism destination, certain tournaments were selected to facilitate business networks with target markets:

We started to promote Dubai in 2011–2012 to establish good relations with China and so we tried to sponsor the best table tennis team. We didn't want it to directly show that it was a commercial effort but we did ask the table tennis federation to come and see what we had. After the press conference, 160 million people saw Dubai and it encouraged Chinese people to come. We now have above 200,000 Chinese people living here. We have dragon mall and a whole city catered to them. We also attracted sports after table tennis such as badminton. It wasn't here as a federation before, but we attracted it and made good relations with the Far East and China. We used the sport as a way of promotion, similar to Emirates Airlines which does a lot with big clubs. Some countries use sports for peace for example; but we use it for networking and commercial and tourism reasons.

Coming straight from a member involved in the selection of sporting events, it was highlighted that smaller scale tournaments that were affiliated with the Far East and particularly China, were used to bridge relations with these target audiences. Dubai had lobbied for events that were more in line with their economic and commercial ambitions. Furthermore, despite the conception that the emirate has strategically chosen niche tournaments that are in line with its affluent touristic profile, such as golf and horseracing (Deloitte, 2015), these events are actually driven by private commercial ambitions rather than nationalistic strategies. Reiterating these commercial ambitions, another candidate used the following example:

The Rugby Sevens is related to Emirates Airlines; they invest in it. Sport is a language. They wanted to talk to Australia and New Zealand so they created Rugby Sevens to communicate with them. They took it as a commercial opportunity because at the end of the day, Dubai uses sport as a commercial approach (D3DSC, April 2, 2018).

As can be seen here, the target audience of Emirates Airlines was not the same as that of Dubai Sports Council. The latter targeted the Far East, whereas the former aimed to establish a relationship with Australia and New Zealand. When speaking to the Head of Strategy at Meydan Holding Company, who was in charge of organising the Dubai World Cup, he noted that although the Sheikh’s passion in horseracing sparked the creation of the tournament, the rulers still view it as a way to drive tourism and the economy. However, falling under the Meydan Company umbrella, the tournament is also ‘used as a catalyst to generate awareness about the Meydan brand’ (D4MH, April 3, 2018). He emphasised that the sport itself does not necessarily need promotion as:

It serves as a brand on its own; it’s like saying I need to promote Christmas. You don’t, it’s a fixed date in the calendar and everybody knows about it. So the race itself helps us promote our brand (D4MH, April 3, 2018).

However, due to the Sheikh’s personal interest in the sport, his inherent nationalistic goals, and the company’s own branding goals, the organising committee finds it challenging to stage the event in a way that works towards all desired outcomes. Highlighting the conflict of interests, one interviewee stated:

The VP is focused on the actual races, the finance guy in our department says leverage the opportunity to make money, whilst others think we should use the event to honour the Sheikh’ (D5MH, April 3, 2018).

Based on this comment, it can be argued that Dubai’s privately-run events are not necessarily geared towards one specific outcome. Instead, they are meant to satisfy the interests of different parties which may diminish their ability to successfully contribute to nationalistic goals.

#### 4.2.3 Providing an entertaining environment for locals

With no reference to a specific nation, the Meydan Horserace does not necessarily aim to establish relationships with specified audiences abroad as was the case with Emirates Airlines’ Rugby Sevens. Instead, it is meant to attract local and international attendees from different social classes to share the Sheikh’s personal passion and promote the company’s brand whilst serving as an entertaining environment.

Like company-held events, members at the Dubai Sports Council reaffirmed that despite continuing to work towards the goals mentioned above, they too have always ensured to host smaller events for the sake of their local population. A busy event schedule was meant to keep locals entertained:

In the beginning, we didn’t try to host big events alone; we encouraged smaller events so that Dubai is constantly an active city. It doesn't need to be that people from South or North America come to us. Even people from the Middle East were our target. So we had around 500 events between five different categories. Local sport events, Gulf events, Arab events, continental events, and international events. We pushed all five tracks. To make sure to incorporate events at all levels to keep Dubai busy. For example, once we had a gymnastic tournament which families went to, at the same time there was a polo tournament which attracted a different audience. So, you provide a variety of sports to cater to all tastes (D3DSC, April 2, 2018).

In addition to this, the Strategic Advisor interviewed at the Executive Council highlighted that:

We did not explicitly work on the sports sector when we worked on the plan. We had other priority sectors that were important and we felt like sport would come in and support the other plans that we had. So if you look through our strategy, you won’t find explicit mention of the sports sector. But sport does have a role to play when looking at the themes. For example, when you talk about experience for example, that’s where sporting events fall. We have beaches and facilities that make Dubai an attractive destination for events and create exciting outings for people to attend.

Thus, similar to the national strategy documents discussed earlier, interview candidates revealed that Dubai’s sport events also work towards ensuring an entertaining local environment that makes Dubai the “preferred place to live, work and visit” and that sport plays a complementary role to attracting international audiences.

#### 4.2.4 Promoting Abu Dhabi as a tourism destination

Four interviews were conducted in Abu Dhabi, three of which were from members of the Abu Dhabi Sports Council (ADSC) and one with an event manager at the Abu Dhabi Formula One. Despite the lack of breadth, these candidates provided valuable insights into Abu Dhabi’s sport strategy which pointed towards the emirate’s soft power ambitions. Interviewees expressed that Abu Dhabi is not well-positioned on the global front as their local neighbours Dubai. Thus, the emirate is using sport to (a) place Abu Dhabi on the map, (b) position it as a touristic destination, and although not explicitly mentioned, (c) compete with Qatar, Dubai and Bahrain through sport.

#### 4.2.5 Place Abu Dhabi on the map

Despite the intrinsic benefits, sport events are primarily used as a marketing mechanism for Abu Dhabi. When speaking to the Head of Marketing and Communications Director at ADSC, he explained that his role consists of managing four departments: Events, Marketing, Communications and Statistics. The mere organisational structure alludes to the way in which the Sports Council strongly associates events with marketing. Furthermore, it was explained that even if events are privately run, they must go through ADSC as all intermissions, media and marketing of the events are handled by the council itself. When asked to explain the constituents of his department, the Director stated:

Let me begin with our most important section which is the events section, all sport events that happen in Abu Dhabi come through us, especially if it is regional, international or under a club…. We help all events including third party events, in terms of their intermissions, media and marketing requirements. All our other departments, they are there to accommodate the events section (AD1ADSC, July 11, 2017).

It can be reasoned that passing all events through a single authoritative body can guarantee a more consistent and coherent approach to market the emirate. As expressed by one candidate, each governmental entity within Abu Dhabi is meant to work towards the broader goals of the emirate, whereas commercial entities are not necessarily obliged to do the same (AD3ADSC, July 11, 2017). Thus, by retaining some media control during third party events, the Council can ensure that these events are being used to promote the emirate rather than being solely used to promote the commercial interests of organising parties.

One respondent expressed that Abu Dhabi has struggled from a lack of visibility on the international front in the past. Dubai has successfully placed itself on the map, independently of the UAE, whereas Abu Dhabi was still buried somewhere within the UAE map. Thus, it has been argued that:

Events are a platform to build Abu Dhabi. It is a worldwide branding initiative. When you pay five minutes to be on Eurosport, it has a big impact on Abu Dhabi. So through hosting events, we have built a name for ourselves which is as strong as, or even stronger than Dubai’s. UAE is already on the map, but we needed to point out what is within this map! People used to associate Abu Dhabi with camels and think that we live in tents… So we needed to create a brand name of the city itself and pull it out of the UAE for them to know us (AD2ADSC, July 11, 2017).

Dubai has managed to successfully separate itself from outdated regional desert-associations by embedding its futuristic infrastructure in the minds of audiences, and now Abu Dhabi intends on doing the same by showcasing itself through sport. Thus, branding the Emirate through these simplistic touristic images, rather than complex explanations and understandings about the intricate culture or policies, have driven Abu Dhabi’s mega-event strategy particularly since the emirate intends to establish itself as a tourism destination.

#### 4.2.6 Position Abu Dhabi as a tourism destination

As was outlined in the document analysis, the interview process also revealed that sports events are used to generate positive economic returns by promoting Abu Dhabi as a touristic destination. Interviewees consistently stressed the importance of showcasing the emirate’s flourishing landscape and touristic attractions through mega-events:

We can’t just do camel races because it’s related to our heritage. Our city can host various types of events. We have the roads to host Abu Dhabi tour for instance. We do not live in a desert where you cannot ride a bike. We have the infrastructure to host these events, we have better roads than Europe which allows us to have a better cycling tour than the one in France… As long as we host outdoor tournaments at the right time during the good weather, there is nothing that can stop us (AD1ADSC, July 11, 2017).

We have developed a lot. The number of hotels and landmarks increased drastically in recent years. Before, if you want to come to the UAE, you would search for a hotel in Dubai. Now you can get the best hotel here. We have pieces of land that were hidden before but now they are being utilised. We have the famous Louvre Museum here and landmarks that Dubai does not even have (AD2ADSC, July 11, 2017).

Collectively, these excerpts frame mega-events as mechanisms for translating Abu Dhabi’s infrastructural modernisation and expanding landmark portfolio into internationally legible destination claims, often in implicit comparison to Dubai. This emphasis on visibility was reinforced through the broadcast logics of mobile events:

With championships like the tour, the filming spans across four hours. It is the only tool to promote our country in this way. You go through the entire landscape…beautiful videos and pictures are shown of our land’ (AD2ADSC, July 11, 2017).

Having heard all the scenic images the nation intended to promote, we specifically inquired whether showcasing cultural elements was of importance to them. It was concluded that modernity rather than culture and tradition, has emerged as the key selling points of the emirate:

Not that much to be honest. The Tourism and Cultural Authority (TCA) are there for this purpose. They can set up their cultural villages within our events but that is not really the main focal point (AD3ADSC, July 11, 2017).

Further pointing towards the synergies between sport events and tourism, interview candidates discussed the various collaborations that take place between the Sports Council and the Tourism and Cultural Authority (TCA). Together, these entities try to prolong the stay of event-related visitors to allow them to experience other non-sport aspects of the Emirate:

Travel packages are created by tourism companies here when events take place. Tourists can choose according to their preferences… Or if we partner with the airlines, we build event-specific packages and market them through the Tourism Authority’s 36 offices which are located in different countries. We try to provide affordable offers and packages whilst maximising the stay of tourists beyond the event itself so they can experience everything Abu Dhabi has to offer (AD2ADSC, July 11, 2017).

Another candidate reiterated these intentions by stating that:

How we accommodate them is we make them feel Abu Dhabi is a nice city and make them want to stay 3 or 4 extra days. When we say we, it’s not just ADSC that does this, it’s the TCA and Abu Dhabi as a whole’ (AD3ADSC, July 11, 2017).

Interviewees also disclosed that each event has a different target audience and thus a distinct marketing approach. International events ‘targeted the whole world’, whereas events such as cricket would target ‘Indians, Pakistanis, South Africans, the British, etc.;’ and camel racing is directed at ‘the Middle East’ (AD3ADSC, July 11, 2017). Again, the responsibility of attracting these tourists is allocated to the tourism authority which is supposed to know how to deal with each target audience:

They know what people are looking for based on demographics. They will not take an Asian and an American to the same place. The Tourism Authority accounts for all the small details relating to each culture’ (AD3ADSC, July 11, 2017).

These comments exhibit the alignment and synergies that exist between sport and tourism within Abu Dhabi. Whilst Dubai used sport as a complementary strategy to their tourism industry, Abu Dhabi is using it as a primary strategy. This is partly attributed to the fact that Abu Dhabi has tried to capitalise on niche segments that Dubai has not in order to redirect tourists from Dubai to Abu Dhabi.

#### 4.2.7 Competing with regional sporting industries

Throughout the interview process, various comments were made in which candidates distinguished Abu Dhabi from its regional counterparts including Dubai, Qatar and Bahrain. As discussed in the previous section, Abu Dhabi’s hotels, dolphins and beaches are up to par with that of its local neighbours, whilst their cultural museums and sport events are considered a unique offering that Dubai does not have.

One interviewee highlighted the intent to establish Abu Dhabi as a sporting hub. This can be seen as direct competition for Qatar which is also working towards attaining the same status:

We have a slogan saying we want to make Abu Dhabi the sport hub of the world mainly through hosting many sports events (A2ADSC, July 11, 2017).

Given that Abu Dhabi has become the second Middle Eastern country to host Formula One, we aimed to better understand how Abu Dhabi distinguishes its race from that of Bahrain to attract a wider audience. One respondent highlighted that Abu Dhabi’s Grand Prix is typically the final race of the season in which the winner is crowned. This in itself can attract a wider audience as Bahrain’s tournament takes place in the beginning of the series. The interviewee also stated that:

More than 60% of spectators came from outside the region’ to attend the Abu Dhabi F1 in 2016, whereas Bahrain’s tournament continuously relies on inviting fans who are mainly local expat workers in the country (AD4F1, July 12, 2017).

Further elaborating on this statement, he explained:

It’s a niche segment there. Somebody invites one person, who invites another and they just go for the sake of the invite and surrounding concerts, not the race itself. Also, Bahrain is a small city. You cannot accommodate all the facilities and tourists… So what we accommodate and what happens around the race is much bigger and better than Bahrain. We have more hotels, boats and yachts that take people around. We have malls, a water park, and soon the Warner Brothers theme park (AD4F1, July 12, 2017).

Once again, the surrounding touristic attractions have been referenced as the key selling point of the Emirate. The way in which the island has developed and the prevalence of entertainment facilities, boats and yachts, are meant to serve as supplementary entertainment options to maximise the stay of those who attend the race. These factors coupled with the crowning of the champion have allowed for the Abu Dhabi Formula One to generate a wider international appeal which competes with Bahrain’s F1.

## 5 Discussion

Accordingly, the contribution of this paper is not to claim that audience agency is a new discovery, nor to replace existing work on soft disempowerment. Rather, the paper synthesises insights from soft power, nation branding, public diplomacy, and sport diplomacy scholarship into a process-oriented framework for analysing how sport-event strategies are articulated, received, and potentially circulated beyond the event itself. In this sense, the Soft Power Cycle is best understood as a heuristic framework that clarifies the movement from strategic intent to familiarity, appreciation, engagement, behavioural uptake, and post-experience diffusion.

### 5.1 Interpreting the divergence between Abu Dhabi and Dubai

Based on the findings obtained from the document analysis and interview process, it was found that Abu Dhabi’s sporting strategies appeared more explicitly outward-looking and centrally linked to soft power ambitions, whereas Dubai’s sport-event landscape appeared more commercially mediated, institutionally dispersed, and only selectively articulated through formal sport-governance strategy in Table 3.

**Table 3:** Summary of findings.

		Dubai		Abu Dhabi	
Conceptual domain	Soft power hierarchy pillars	Doc analysis	Interview analysis	Doc analysis	Interview analysis
Soft power	Attract tourism	☑ Continue to attract tourists, complemented by sport tourism	☑ Complement existing tourism profile	☑ Attract niche tourist segment that has been neglected by Dubai (focus on sport and culture)	☑ Use sport to distinguish tourism profile from Dubai, target affluent spenders
	Attract investment/Trade/Business	☑ Attract business, investment and trade	☑ Commercial partnerships	☒ N/A	☒ N/A
	Obtain political support	☒ N/A	☒ N/A	☒ N/A	☒ N/A
Public diplomacy	Establish long-lasting relationships	☒ N/A	☑ Business partnerships for commercial entities	☒ N/A	☒ N/A
	Build trust/Credibility	☑ Secure internal foundations to make Dubai preferred destination to visit, work and live	☒ N/A	☒ N/A	☒ N/A
	Cultivate understandings	☒ N/A	☒ N/A	☒ N/A	☑ Showcase Abu Dhabi’s scenic landscape, cultural and sporting attractions
Nation branding	Establish competitive identity	☑ Already established as tourism hub, now focused on global economic hub status	☑ Previously used sport to reaffirm tourism status	☑ Tourism and Cultural hub	☑ Cultural and sporting hub to attract tourism
	(Re)mold Image/Identity	☑ Advanced nation	☑ Previously used to promote new beaches	☑ Globally open economy, cultured	☑ Modernised, scenic landscape
	Increase awareness	☒ N/A	☒ N/A	☑ Increase awareness of Abu Dhabi	☑ “Pull Abu Dhabi out of the UAE Map”

Source: author’s own work.

Whilst one might assume that the less centrally state-led nature of Dubai’s strategies contradict the overarching aim of this research, they actually reaffirmed the theoretical underpinnings of this study by exemplifying how states that are striving to emerge from the shadows of its local/regional neighbours are using sport events for such measures, whereas those that are globally recognised and well-positioned (Dubai) are not.

Despite similar external ambitions, Abu Dhabi had a more fragmented approach in which explicit references were made to branding the emirate and positioning it on the map via sport events but not to foster deeper dialogues or enhance the emirate’s credibility. Instead, the niche segments of sport and culture were being used to distinguish Abu Dhabi’s tourism profile from that of its local neighbours Dubai and sport events were strategically selected based on their ability to showcase the emirate’s scenic landscape and to attract international audiences and affluent spenders. Interviewees also alluded to the competitive nature of their sport event strategy by highlighting how their Formula One was different to that of Bahrain’s, a pattern that reflects wider regional competition around sport, visibility, and geopolitical positioning in the Gulf (Bianco and Sons, 2023; Ulrichsen, 2023; Brannagan and Reiche, 2025). This competitive stance reaffirms the way in which sport events are used to signify and symbolise the “power status” of emerging nations.

Although Dubai’s sport strategy was found to be less centrally state-led than that of its neighbours, interview candidates in Dubai explained that sport events were previously used for external reasons in the past. Prior to cementing itself as a tourism hub, sport events were strategically selected to showcase Dubai’s beaches to help facilitate the growth of its tourism industry or to establish networks with target markets such as China. However, now that the emirate is well-positioned on the international front, the emirate’s events are primarily geared towards providing an entertaining environment for locals and tourists, whilst those that are run by corporate entities are used to pursue commercial rather than national interests. Whilst Abu Dhabi have used sport as anchor strategies embedded within national visions, Dubai appears to position sport less as a standalone state-led soft power instrument and more as a complementary mechanism embedded within its wider tourism, commercial, leisure, and lifestyle ecosystem.

It is important, however, not to interpret Dubai’s comparatively less centrally state-led orientation as evidence that sport lacks external visibility or soft power relevance in the emirate. Dubai hosts several internationally recognised sport events, including commercially organised tournaments and privately or semi-privately managed events with substantial international media reach. The distinction advanced here is therefore not that Dubai’s sport-event landscape is disconnected from soft power effects, but that these effects appear to be less explicitly coordinated through the formal strategic remit of the Dubai Sports Council than in Abu Dhabi’s case, echoing recent work on how sport-related visibility can be produced through corporate, commercial, and semi-state channels rather than direct state strategy alone (Chadwick and Anagnostopoulos, 2024; Crossley and Woolf, 2024; Grix et al., 2025). In Dubai, sport often operates through a more decentralised ecosystem involving corporate sponsors, tourism actors, airlines, venue operators, and ruling-family-linked entities. These events may still reinforce Dubai’s global image as a commercial, leisure, and lifestyle hub, but they do so through market-facing and brand-supporting logics rather than through a single, centrally articulated sport-as-soft-power strategy. This suggests that Dubai’s sport-event strategy should be understood not as non-soft-power-oriented, but as commercially mediated and institutionally dispersed.

### 5.2 Strategic maturity and the instrumental role of sport

A clear sense of advancement and modernity was provided by Dubai’s built environment which consisted of the most lavish, luxurious and largest developments in the region including the airport, roads, residential buildings, office towers, hotels, malls and manmade islands. In addition to these elements, Dubai’s touristic attractions and the prevalence of foreign tourists was a clear indication of the emirate’s international tourism hub status. Abu Dhabi’s built environment also consisted of lavish developments, but on a smaller and more dispersed scale than Dubai. Despite the cultural museums and entertainment options such as theme parks, it became evident why Abu Dhabi has tried to capitalise on sport and culture as niche segments to deter tourists from Dubai’s thriving environment. Although Abu Dhabi is the capital of the UAE, Dubai was more progressive and evidently more recognised as the global tourism hub.

When evaluating each emirate’s sporting environment, it was found that Abu Dhabi’s Formula One was most successful in attracting international tourists. The event was also strategically positioned alongside theme parks to provide tourists with additional entertainment options and strategically managed to showcase Emirati culture by placing cultural displays throughout open areas. These observations signify the synergies between Abu Dhabi’s national objectives and sport strategies which are highly driven by its tourism aspirations. Despite hosting the same event, it was surprising to find the extent to which Bahrain’s Formula One was different to that of Abu Dhabi’s. Not only were vacancy rates significantly higher in Bahrain, but rather than comprising of international audiences, attendees were primarily non-Bahraini residents, which indicated a lack of sporting culture amongst Bahrainis themselves. Thus, cultural and touristic elements were not promoted via the event as entertainment options were highly geared towards younger demographics.

Dubai’s largest sport events were highly catered towards the local population but also to target audiences of the organising entities themselves such as those from New Zealand and Australia in the case of the Rugby Sevens, or the Sheikh himself in the case of the Dubai World Cup, reflecting the importance of institutional agility, stakeholder literacy, and adaptive commercial strategy in emerging economy contexts (Alainati et al., 2025; Alsabah et al., 2025). Due to the commercialization of events, it was difficult to pinpoint any national ambitions based on the way these tournaments were staged. Furthermore, based on the lack of branding, the lack of advertising and the lack of interest of attendees in the sport event itself, it became evident that sport was not a primary anchor for the emirate nor for those attending. Instead, it served as a “side-event” for Dubai’s existing environment and for those seeking to socialise or entertain corporate guests.

### 5.3 Advancing the soft power framework: from hierarchy to cycle

Soft power is better conceptualised as a cyclical process rather than a linear sequence, particularly in light of recent work that treats influence as contingent on credibility, reception, and the interaction between power resources and audience interpretation (Nye, 2021; Gallarotti, 2021; Gallarotti, 2022; Santos, 2021; Henne, 2022). Once a state’s nation branding and public diplomacy efforts have contributed to behavioural uptake, the audience becomes an additional site of influence. Visitors who encounter the host nation through sport events may communicate their experiences through interpersonal networks and digital platforms, thereby shaping wider perceptions through forms of informal endorsement. This feedback loop means that soft power outcomes can intensify when lived experience reinforces projected narratives or deteriorates when experience contradicts them. For this reason, evaluating soft power requires attention not only to message production, but also to how post experience communication sustains, modifies, or destabilises reputational effects over time.

**Figure 3:**
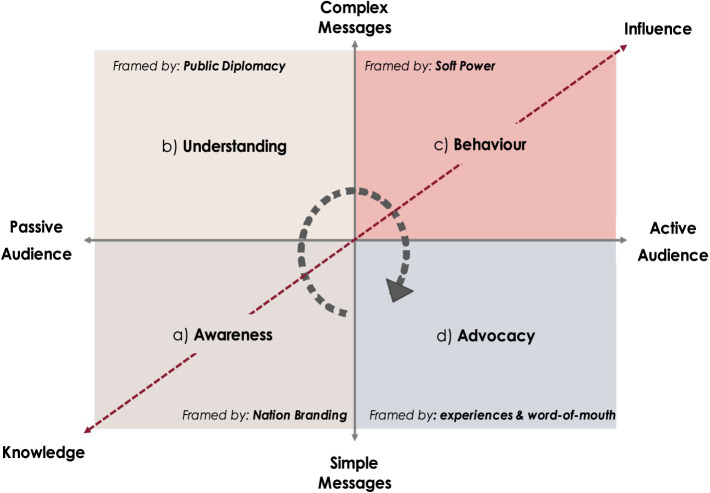
Soft power cycle source: author’s own work.

Based on these understandings, the soft power hierarchy may be advanced in the following manner as shown in Figure 3:

Figure 3 summarises the Soft Power Cycle by mapping how nation branding and public diplomacy operate as preconditions for behavioural influence. Quadrant (a) captures early-stage influence through nation branding, where audiences are primarily passive recipients of simplified messages that build recognition and initial positive associations. Quadrant (b) reflects public diplomacy as a deeper communicative phase that can cultivate more textured understandings and relational credibility, although audiences may still remain passive in the sense that they are not yet behaviourally committed.

The model then shifts attention from communication to uptake and diffusion, which in the UAE cases can be seen in the emphasis on converting event visibility into tourism, commercial activity, and wider reputational circulation. Quadrant (c) represents the point at which audiences convert favourable perceptions into voluntary behaviour, such as visiting, investing, trading, endorsing, or offering political support. The transition from appreciation to behavioural uptake depends on several conditions, including the perceived credibility of the host, the alignment between projected messages and lived experience, the relevance of the event to target audiences, and the availability of practical pathways for action, such as travel packages, media circulation, investment opportunities, or institutional partnerships. Quadrant (d) conceptualises what happens after behaviour occurs, where first-hand experience can be communicated to wider publics through interpersonal networks and digital platforms, thereby amplifying or weakening the original message. The core contribution of the cycle is therefore its emphasis on feedback. Audiences do not only respond to soft power strategies, they can also extend, reshape, or destabilise them through post-experience communication.

To ground the model in the UAE cases, each quadrant can be linked to the empirical findings. Quadrant (a), which concerns familiarity, is reflected most clearly in Abu Dhabi’s use of sport events to “place Abu Dhabi on the map” by showcasing its name, landscape, infrastructure, and destination image through internationally broadcast events. Dubai also demonstrates this stage, particularly in earlier efforts to use beach football and other events to increase recognition of its beaches and tourism assets. Quadrant (b), which concerns appreciation, is evident in the way Abu Dhabi sought to move beyond visibility by associating events with modernity, competence, hospitality, and tourism quality, whilst Dubai used sport to reinforce its image as a liveable, commercially vibrant, and entertainment-rich city. Quadrant (c), behavioural uptake, is reflected in the emphasis placed by both emirates on attracting visitors, extending tourist stays, generating commercial activity, and encouraging international engagement around events. Quadrant (d), post-experience diffusion, is less directly measured in this study because audience-side data were not collected, but it is implied in stakeholder accounts that visitors’ experiences, media coverage, and event-related imagery can circulate beyond the event itself and shape wider perceptions of the host destination.

The Soft Power Cycle builds directly on the soft disempowerment literature, which has shown that sport can undermine rather than enhance attraction when projected narratives are contradicted by international scrutiny, credibility gaps, or negative audience interpretation (Brannagan and Giulianotti, 2015, 2018). Soft disempowerment is therefore not outside the cycle, but represents a possible negative pathway within it. The cycle can generate positive reputational circulation when lived experience reinforces projected narratives, but it can also generate reputational reversal when audiences, media actors, or advocacy groups interpret sport-event strategies as inauthentic, manipulative, or misaligned with host-state practises (El-Nawawy and Elmasry, 2024; Karlsson and Wenell, 2026).

Whilst other factors may frame each of the elements above, the model is meant to depict the *intentional* and *active* involvement of *nations* and *audiences* which can lead to *positive* outcomes. Thus, it does not account for the soft disempowerment cycle which typically arises from second-hand sources, national tragedies, historical occurrences, the unintentional misalignment in a nation’s cultural norms, political values, or policies, nor the negative first-hand experiences and subsequent bad-mouthing which can lead to rejection, resentment and resistance from international audiences.

## 6 Limitations and future research

This study has several limitations that should be acknowledged. The study is based on a relatively small qualitative sample of 11 interviews. Although the participants were selected because of their direct involvement in sport governance, event organisation, and strategic planning, the sample does not capture the full range of institutional, commercial, civic, and audience perspectives that shape sport-event outcomes. The findings should therefore be understood as analytically interpretive rather than statistically generalisable. The purpose of the study is not to measure the prevalence of particular views across the UAE sport sector, but to develop a contextualised understanding of how key stakeholders interpreted the strategic role of sport events during the period under study.

The analysis relies primarily on elite and institutional stakeholder perspectives. This provides valuable insight into strategic intent, policy rationales, and event-selection logics, but it also means that the study is weighted towards those involved in designing, governing, or implementing sport-event strategies. As a result, the paper cannot fully assess how these strategies were received by international tourists, residents, athletes, sponsors, media actors, or wider foreign publics.

A further limitation concerns the institutional lens through which Dubai’s sport-event landscape is interpreted. Several of Dubai’s most internationally visible sport events operate outside the direct strategic remit of the Dubai Sports Council and are shaped by corporate, commercial, tourism, airline, or ruling-family-linked actors. As a result, the study may capture the formal sport-governance perspective more fully than the broader ecosystem of commercially organised events that also contribute to Dubai’s international visibility. This limitation is especially relevant because events outside DSC’s direct purview may still generate soft power effects by reinforcing Dubai’s image as a global leisure, business, and lifestyle destination. Future research should therefore examine Dubai’s sport-event ecosystem across a wider range of institutional actors, including event owners, sponsors, tourism authorities, broadcasters, airlines, and international audiences.

The study does not include audience-side data. This is particularly important because the Soft Power Cycle proposed in this paper conceptualises audiences as active agents who may reinforce, reshape, or undermine soft power through their experiences and subsequent communication. Whilst the model highlights this process theoretically, the empirical data do not directly measure audience perceptions, behavioural responses, or post-event communication. Future research could address this limitation by incorporating surveys, interviews, digital trace data, media analysis, or social media analysis to examine how different audiences interpret and circulate sport-event experiences.

The Abu Dhabi interview sample is also institutionally concentrated, with three participants drawn from the Abu Dhabi Sports Council and one from Formula One event management. This means that the Abu Dhabi findings are especially reflective of sport-governance and event-management perspectives rather than the full range of actors involved in Abu Dhabi’s sport-event ecosystem. This imbalance affects the comparative claims by making the Abu Dhabi case more strongly grounded in one institutional viewpoint than the Dubai case. Accordingly, the study does not treat the Abu Dhabi interviews as exhaustive evidence of the emirate’s overall strategic posture. Instead, they are interpreted alongside documentary sources as indicative of how key institutional actors framed sport events during the period under study.

Finally, the comparative design is limited to Abu Dhabi and Dubai. This focus allows for an in-depth comparison of two sub-national actors within the same federal context, but it limits the extent to which the findings can be generalised to other emirates, Gulf states, or sport-event systems. Future research could extend this framework by examining more recent sport-event developments in the UAE and Saudi Arabia, comparing pre- and post-COVID event strategies, or testing the Soft Power Cycle across different regional and institutional contexts.

## 7 Conclusion

This study examined how sport events are mobilised as instruments of soft power, nation branding, and public diplomacy within the UAE through a comparative analysis of Abu Dhabi and Dubai. The findings show that sport does not operate as a uniform or automatically effective pathway to influence. Rather, its strategic value depends on institutional intent, reputational maturity, governance arrangements, and the wider positioning of the actor deploying it. Abu Dhabi appeared to use sport events more explicitly as an outward-facing strategy aimed at increasing international visibility, strengthening tourism positioning, and competing for attention in a crowded regional environment. Dubai, by contrast, appeared to use sport less as a centrally coordinated soft power instrument and more as a commercially mediated and institutionally dispersed mechanism embedded within its wider tourism, leisure, lifestyle, and global brand ecosystem. These differences highlight the importance of examining sub-national variation when analysing sport, soft power, and nation branding in federal and non-Western contexts.

The study contributes to soft power scholarship by positioning influence as a dynamic and contingent process rather than a linear sequence of communication and response. The Soft Power Cycle developed in this paper synthesises insights from nation branding, public diplomacy, sport diplomacy, and soft disempowerment by showing how sport-event strategies may be reinforced, interrupted, or reversed through audience reception, credibility, lived experience, and post-experience circulation. The findings also suggest that second-order and recurring sport events deserve greater attention, as they may offer sustained opportunities for visibility, engagement, and reputational positioning when aligned with broader strategic objectives. For policymakers, this underscores the need to approach sport-event hosting as a coordinated, adaptive, and context-sensitive strategy rather than as an automatic route to international influence.

## Data Availability

The raw data supporting the conclusions of this article will be made available by the authors, without undue reservation.
