# Supplementary material for: Entrepreneurial nations competing for attention: sport mega-events as strategic assets for soft power
Source: Front Sociol. 2026 Jun 25;11:1811928. doi: 10.3389/fsoc.2026.1811928 (PMC13345936; doi:10.3389/fsoc.2026.1811928)
Supplement: Supplementary file 1 [file Table_1.DOCX]

# Appendix A. Documentary Corpus Reviewed for Dubai and Abu Dhabi

| **Case** | **Document reviewed** | **Source / issuing body** | **Year** | **Document type** | **Purpose in analysis** |
| --- | --- | --- | --- | --- | --- |
| Dubai | Dubai Strategic Plan 2015 | Dubai Executive Council | 2007 | Government strategy / vision | Provided the baseline for Dubai’s development priorities and early positioning as a major city, business destination, and tourism-oriented economy. |
| Dubai | Dubai Plan 2021 | Dubai Executive Council | 2014 | Government strategy / vision | Used to identify Dubai’s post-2015 priorities around global economic hub positioning, liveability, tolerance, and destination attractiveness. |
| Dubai | Dubai Plan 2021 Strategic Report / Outcomes Report | Dubai Executive Council | 2017 | Government progress report | Used to assess how Dubai reported progress against its strategic objectives and framed international events, conferences, tourism, and landmark projects. |
| Dubai | Dubai Sports Council Strategic Plan 2007-2010 | Dubai Sports Council | 2008 | Sport-sector strategy | Used to examine Dubai’s early sport-sector priorities, including club development, professionalisation, and the role of sport within the emirate’s internal development agenda. |
| Dubai | Dubai Sports Council Strategic Plan 2011-2015 | Dubai Sports Council | 2012 | Sport-sector strategy | Used to identify the relationship between sport, tourism profile, sport awareness, investment, and local participation objectives. |
| Dubai | Dubai Sports Council Unveils 2016-2020 Strategy | Gulf News | 2016 | Authoritative media report / official interview | Used because the full 2016-2020 sport strategy was not publicly available; provided supplementary insight into the continued direction of DSC strategy. |
| Dubai | Economic Impact of Sport in Dubai | Deloitte / Dubai Sports Council | 2015 | Industry / commissioned report | Used to contextualise Dubai’s sport-event economy, commercial event portfolio, and the economic role of sport within the wider tourism and business ecosystem. |
| Abu Dhabi | Abu Dhabi Plan / 2030 Vision materials | General Secretariat of the Executive Council / Abu Dhabi Government | 2008 | Government strategy / vision | Used to identify Abu Dhabi’s long-term development aims, including the objective of building a competitive, sustainable, and globally open economy. |
| Abu Dhabi | Abu Dhabi Economic Vision 2030 | Department of Economic Development / Government of Abu Dhabi | 2008 | Economic development strategy | Used to examine Abu Dhabi’s economic diversification, tourism development, and positioning of leisure, culture, and sport within the emirate’s development strategy. |
| Abu Dhabi | Abu Dhabi Policy Agenda 2007-2008 | Abu Dhabi Executive Council | 2008 | Government policy agenda | Used as contextual material for Abu Dhabi’s policy priorities and governance approach during the period leading into its sport-event expansion. |
| Abu Dhabi | Abu Dhabi Tourism and Culture Authority visitor/tourism report / Tourism Economic Review | Abu Dhabi Tourism and Culture Authority / Abu Dhabi Council for Economic Development | 2014 | Tourism and economic report | Used to assess Abu Dhabi’s tourism positioning, target markets, destination-branding aims, and the role of events in generating international visibility. |
| Abu Dhabi | Sport in Abu Dhabi to Receive a Boost | Gulf News | 2008 | Authoritative media report / official interview | Used because official Abu Dhabi sport strategies were not fully publicly available; provided insight from senior sport officials on the emirate’s event-hosting rationale. |
| Abu Dhabi | The Report: Abu Dhabi 2016, tourism, culture, and sport sections | Oxford Business Group | 2016 | Industry report / official interview material | Used to supplement missing sport-strategy documents and provide contextual detail on Formula One, golf, cycling, sailing, air racing, and sport-tourism positioning. |

*Source: Authors’ own work*
